# Prevalence and Determinants of Plastic Surgery Among Adults in Saudi Arabia

**DOI:** 10.7759/cureus.52036

**Published:** 2024-01-10

**Authors:** Amr Arkoubi, Faris Aldaghri, Wael A Daghstani, Tamara A Hafiz, Ghaida B Alanazi, Alwaleed I Almughira, Shahad AlShehri, Maram K Alshammari, Reemah AlQahtani

**Affiliations:** 1 Department of Plastic and Reconstructive Surgery, Imam Mohammad Ibn Saud Islamic University, Riyadh, SAU; 2 Faculty of Public Health and Health Informatics, Umm Al-Qura University, Makkah, SAU; 3 Faculty of Medicine, University of Tabuk, Tabuk, SAU; 4 Department of Medicine and Surgery, King Faisal University, Al Ahsa, SAU; 5 Department of Plastic and Reconstructive Surgery, King Khalid University, Abha, SAU

**Keywords:** saudi arabia, image, self-esteem, perception, aesthetic surgery, cosmetic surgery, plastic and reconstructive surgery

## Abstract

Background: Social and cultural factors have a significant impact on body image standards, and the media's messages play a crucial role in shaping beauty ideals. People's perceptions of beauty can be negatively affected by social media, which promotes unrealistic beauty standards and can lead to a desire for plastic surgery. Plastic surgery includes both reconstructive and aesthetic procedures and has become increasingly popular worldwide. In Saudi Arabia, there has been a significant increase in the number of women seeking cosmetic surgery, particularly breast augmentation, rhinoplasty, and liposuction, indicating that cosmetic surgery is becoming more accepted as a way to enhance beauty.

Aims: To investigate the frequency of performance of plastic and aesthetic surgical procedures among adults aged 18 to 60 years old in Saudi Arabia, as well as the determinants that may contribute to it.

Methodology: A cross-sectional web survey with a representative sample (n = 3238) of individuals in Saudi Arabia was carried out from 10 April 2023 to 28 October 2023.

Results: The study included a cohort of 3238 participants, with 1328 individuals choosing to undergo plastic surgery. Among the surgical subgroups, the predominant procedure of choice was breast augmentation, representing 1009 cases (31.2%), while a minority opted for alternative plastic surgical interventions. Notably, the primary impetus behind plastic surgery, as indicated by the majority of participants (38.4% of the overall 41% who underwent the procedure), was appearance enhancement. Examination of the participants' demographic profile revealed a predominance of women between the ages of 18 and 30, married individuals, predominantly having a primary level of education, with a subset working in the public sector. Furthermore, a considerable segment of participants (32.6%) indicated low income, while 31.8% fell into the category of obesity.

Conclusion: To address the multifaceted nature of plastic surgery decisions in Saudi Arabia, the key focus should be on promoting cultural acceptance, creating a supportive environment, and establishing ethical guidelines. This entails conducting awareness campaigns, promoting inclusive beauty standards, facilitating community discussions, fostering collaboration with support organizations, integrating mental health services, and ensuring rigorous monitoring of safety and professionalism in plastic surgery practices. By implementing these recommendations, individuals seeking plastic surgery can enhance their overall well-being and satisfaction.

## Introduction

Sociocultural theories have brought attention to the impact of societal and cultural factors on the formation of body image standards [1,2]. Research has shown that media messages play a significant role in shaping individuals' perceptions of beauty ideals, leading to negative effects resulting from social comparisons [3,4]. Additionally, popular social networking sites (SNS) like Instagram and TikTok offer users the ability to instantly modify their appearance and conform to unrealistic beauty standards [2]. Moreover, the availability of editing software can distort individuals' self-perception, fostering a desire for plastic surgery as a solution to perceived imperfections [5-7]. Several studies conducted between 2019 and 2021 have suggested a positive association between the use of social media, including engagement with SNS, and a greater acceptance of plastic surgery [8-12].

Plastic surgery, as a distinct surgical subspecialty, encompasses a range of procedures intended to improve either the functionality or appearance of various body components through reconstructive or cosmetic operations [13]. Examples of surgical procedures within this field include blepharoplasty, rhinoplasty, breast augmentation, as well as non-surgical interventions such as botulinum toxin injections [14]. Plastic surgery can be further classified into two subspecialties: cosmetic surgery (CS) and reconstructive plastic surgery. While reconstructive surgery primarily focuses on restoring function, cosmetic surgery aims to enhance an individual's esthetic appeal. It is important to note that while the primary objective of reconstructive plastic surgery is functional restoration, efforts may also be made to achieve a person's characteristic appearance [15]. The term "reconstructive surgery" is commonly used to describe this particular subspecialty within the field of plastic surgery [16].

The primary objective of plastic surgery is to address and rectify issues related to damaged, lost, ill, deformed, or malformed body areas through surgical interventions. This often involves procedures such as tissue transplantation, where tissue is transferred from one part of the body to another, such as the skin or cartilage. Over time, the purpose of plastic surgery has expanded to encompass cosmetic goals and the reversal of aging effects. This expansion can be attributed to the substantial influence of beauty standards in contemporary society [17]. Notably, there has been a significant global increase in the prevalence of plastic surgery over the past decade [13]. This upsurge in plastic surgery prevalence suggests a noteworthy shift in attitudes towards and acceptance of plastic surgery.

Based on the International Society of Aesthetic Plastic Surgery (ISAPS) Global Statistics for 2020 report, the United States, Brazil, Mexico, Germany, and Colombia ranked as the top five countries in terms of the highest number of plastic surgery procedures performed. The top five plastic surgery procedures conducted in 2020 were liposuction, breast augmentation, eyelid surgery, abdominoplasty, and rhinoplasty [18].

In the Kingdom of Saudi Arabia, plastic surgery has gained popularity in recent years, with a significant number of procedures being performed annually. In 2020 alone, there were 24,964 plastic surgery procedures conducted in the country [18]. The most sought-after procedures among women aged 35 to 50 were breast augmentation, liposuction, and eyelid surgery, while men commonly underwent gynecomastia, eyelid surgery, and liposuction [18]. Both genders showed a preference for non-surgical treatments such as hyaluronic acid, hair removal, and botulinum toxin [18]. It is important to note that trends in plastic surgery can vary over time and across different geographical areas [18]. Among the top 30 nations globally with the highest rates of plastic surgery, Saudi Arabia is ranked 29th [19].

The Kingdom of Saudi Arabia has witnessed a significant surge in the number of female patients seeking cosmetic surgery, highlighting a growing trend towards using plastic surgery for beauty enhancement. This trend is particularly noticeable among women, with a lesser extent of interest among males [20]. Media coverage of plastic surgery outcomes has proven influential, shaping the decisions of individuals who visit plastic surgery clinics [14]. Notably, individuals with low self-esteem tend to exhibit a higher acceptance rate for plastic surgery [8]. Additionally, external motivators like the desire to avoid racial bias and the fear of age discrimination, along with internal motivators such as depression, shyness, and social anxiety, contribute to individuals' consideration of plastic surgery [8]. Research has also explored the impact of socioeconomic factors on the prevalence of plastic surgery. Demographic variables, including age, gender, economic status, and educational level, have been identified as influential factors in individuals' decisions to undergo plastic surgery [21].

To the best of the authors' knowledge, previous research has primarily focused on investigating the impact of social media on individuals' inclination to undergo plastic surgery, leading to a gap in knowledge regarding the influence of other variables, such as self-esteem and socioeconomic factors. In light of this gap, the present study aimed to explore the prevalence of plastic surgery among both women and men in the Kingdom of Saudi Arabia while also examining the potential determinants associated with the procedure. Specifically, the study sought to investigate the impact of body image, self-esteem, acceptance of cosmetic surgery (CS) scores, and the influence of pictures featuring friends or celebrities on the desire to undergo plastic surgery. Given the escalating demand for plastic surgery in the Kingdom of Saudi Arabia, it is crucial to identify the underlying factors that motivate individuals to seek such procedures. By examining a range of variables beyond the scope of social media, this study contributes to a more comprehensive understanding of the multifaceted factors that influence individuals' decisions to undergo plastic surgery. The findings of this study may prove beneficial for healthcare professionals and policymakers in developing appropriate interventions and formulating policies to address the increasing demand for plastic surgery in the region.

Aim of the study

The aim of this study was to examine the prevalence of plastic surgery among adults aged 18-60 years in Saudi Arabia and identify the determinants associated with its occurrence. Specifically, the objectives were to determine the prevalence of plastic surgery in the adult population (18-60 years) of Saudi Arabia and investigate the potential role of various covariates, including sociodemographic factors, history of previous plastic surgery, body mass index (BMI), self-perceived body and facial images, influence of friends' and celebrities' appearance, Appearance Comparison, Acceptance of Cosmetic Surgery Scale (ACSS) scores, Body Appreciation Scale (BAS) scores, and Rosenberg Self-Esteem Scale scores, as determinants of plastic surgery prevalence. By addressing these objectives, this study aimed to provide comprehensive insights into the prevalence and underlying determinants of plastic surgery in the Saudi Arabian adult population.

## Materials and methods

The prevalence of plastic surgery and its determinants were assessed using a structured cross-sectional web-based survey conducted from April 10, 2023, to October 28, 2023. The study encompassed all regions of the Kingdom of Saudi Arabia, including the Central, Northern, Eastern, Western, and Southern regions. The survey initially involved 3452 respondents, and after applying exclusion criteria, a representative sample of 3238 individuals aged between 18 and 60 years, from the general population of Saudi Arabia, was formed.

According to the Raosoft sample size calculator available at the online Raosoft website, the minimum recommended sample size is 385 based on the given conditions. These conditions include a total adult population in Saudi Arabia (aged 18 to 60 years) of 23,735,248, a response distribution of 50%, a confidence level of 95%, and a margin of error of 5%. However, to mitigate bias and increase the reliability of the survey results, it is advised to double the sample size to 770.

Inclusion criteria

The study included participants who were part of the general population aged between 18 and 60 years old and residing in the Kingdom of Saudi Arabia.

Exclusion criteria

Participants who fell under the following criteria were excluded from the study: individuals below the age of 18 or above the age of 60 years and those who did not reside in the Kingdom of Saudi Arabia.

Data collection tools

Data for the study was collected using structured self-questionnaires written in Arabic. The completion time for the questionnaire was estimated to be around five to eight minutes. The questionnaire consisted of three sections.

The first section focused on obtaining participants' consent to participate in the research. It also included sociodemographic questions to gather information about age, gender, nationality, region, social status, occupation, education, and income.

The second section of the questionnaire addressed plastic surgery history. Participants were asked whether they had a history of cosmetic or plastic surgery. If they answered positively, they were further asked to specify the type of surgery and the reasons or determinants behind their decision, which could include improving appearance due to self-image, friends' image, celebrity image, or medical reasons. Additionally, participants' body mass index (BMI) was assessed in this section.

The third and final section of the questionnaire included three widely used scales. The first scale was the Acceptance of Cosmetic Surgery Scale (ACSS), which aimed to measure participants' acceptance of cosmetic surgery [22]. The second scale used was the Body Appreciation Scale (BAS), designed to assess body appreciation [23]. Finally, the Rosenberg Self-Esteem Scale (RSES) was employed to measure participants' self-esteem levels [24]. These scales were included to gather relevant data on participants' attitudes towards cosmetic surgery, body appreciation, and self-esteem.

Additional information

Self-images are how individuals perceive and emotionally represent their own physical appearance, abilities, and personality traits, including body size, shape, weight, and overall identity.

Images of friends are an individual's mental representation of their friends' physical appearance and personality traits, which can influence their own self-image and beauty standards.

Images of celebrities are an individual's mental representation of well-known individuals' physical appearance and personality traits, often portrayed through media, which can impact an individual's perception of beauty and contribute to unrealistic beauty standards.

Acceptance of Cosmetic Surgery Scale (ACSS) 

The Acceptance of Cosmetic Surgery Scale (ACSS) is a 15-item questionnaire that measures an individual's acceptance of cosmetic surgery. It consists of three subscales capturing different aspects of acceptance related to cosmetic procedures. Participants rate each item on a seven-point Likert scale, with higher scores indicating a greater inclination to accept cosmetic procedures. The scale has demonstrated good reliability (Cronbach's α = 0.88 during development and α = 0.93 in the study). Based on the scoring system, the scores were categorized into three groups: low acceptability, moderate acceptability, and high acceptability. The scoring range ranged from a minimum possible score of 15 (if the respondent chose "strongly disagree" for all 15 questions) to a maximum possible score of 105 (if the respondent chose "strongly agree" for all 15 questions). Each participant's total score was calculated, and cutoff points were set at approximately 35 and 70 by dividing the scoring range into three equal intervals. Accordingly, participants were assigned to the respective category based on their scores. Scores below 35 indicated low acceptance, scores between 35 and 70 represented moderate acceptance, and scores above 70 indicated high acceptance.

The Body Appreciation Scale-2 (BAS-2)

The Body Appreciation Scale-2 (BAS-2) is a 10-item scale used to measure body appreciation. Participants rate each item on a five-point Likert scale, ranging from one (never) to five (always). Higher scores on the scale indicate greater levels of body appreciation. The total score is obtained by summing the responses, resulting in a score between the minimum possible score of 10 and the maximum possible score of 50. According to previous research, the BAS-2 has been found to exhibit a unidimensional factor structure, indicating that it measures a single construct. The scale has demonstrated strong internal consistency (Cronbach's α = 0.97) and good construct validity and test-retest reliability (r = 0.90) in various samples of men and women from community and college settings. Additionally, in another study, the BAS-2 showed excellent internal consistency (Cronbach's α = 0.954) and McDonald's ω value of 0.956, indicating high reliability. These findings support the scale's reliability and validity as a measure of body appreciation in the context of the current study [23]. To interpret the BAS-2 scores, the authors established grading divisions. Scores ranging from 10 to 25 indicate low body appreciation, suggesting that individuals in this category have relatively low levels of body appreciation. Scores from 26 to 35 represent moderate body appreciation, indicating that individuals in this category exhibit moderate levels of body appreciation. Scores from 36 to 50 indicate high body appreciation, suggesting that individuals in this category demonstrate high levels of body appreciation.

Rosenberg Self-Esteem Scale (RSES)

The Rosenberg Self-Esteem Scale is a widely used self-report questionnaire that measures the level of self-esteem in both women and men. The scale consists of 10 items that assess an individual's feelings towards themselves, with an emphasis on overall self-worth and self-acceptance. The items are rated on a positive and negative scale. The psychometric properties of the Rosenberg Self-Esteem Scale have been found to be strong, including excellent internal consistency, test-retest reliability, and construct validity. It has been employed in numerous studies to examine the relationship between low self-esteem and conditions such as depression, anxiety, and stress. Furthermore, the scale has been utilized in clinical settings to explore the role of self-esteem in various mental health issues. The scoring system for the scale involves using a Likert scale with ten questions. Responses are assigned a score of one for "strongly disagree," two for "disagree," three for "agree," and four for "strongly agree." However, questions two, five, six, eight, and nine are reverse scored. Total scores are then calculated based on the scale. Participants who obtain high scores are identified as having a high level of self-esteem. To interpret the scores, the authors of the scale categorized them into three distinct groups: low self-esteem, average self-esteem, and high self-esteem. Scores below 20 indicate low self-esteem, scores ranging from 20 to 30 suggest average self-esteem, and scores above 30 indicate high self-esteem. This categorization allows for an understanding of an individual's level of self-esteem based on their total score on the scale.

Data management

The extracted data were examined and encoded using IBM SPSS version 22 (SPSS, Inc., Chicago, IL). Demographic information, plastic surgery-related components, and plastic surgery-related determinant factors were then subjected to descriptive analysis in the form of frequency and percentage. The chi-squared test was used to calculate the P-value, and a P-value of 0.05 or less was considered statistically significant. The study's findings were presented using tables and graphs.

Ethical consideration

The study received ethical approval from the Bioethics Committee at Imam Abdulrahman Bin Faisal University (IAU) on September 11, 2023. The ethical approval number for the study is HAPO-01-R-011. In order to ensure participants' informed consent, a cover letter providing information about the study was presented to them online. Participants were explicitly informed of their right to decline participation at any time without the need to provide a reason.

## Results

A total of 3238 participants were enrolled in the study. The majority of participants were Saudi (97.0%) and from the central region (1479, 45.7%). More than half of the individuals were aged between 18 and 30 years (2177, 67.2%) and were married (2055, 63.5%). Furthermore, 1586 (49.0%) of respondents were employed in the public sector, approximately 1430 (44.2%) had a bachelor's degree, and 1015 (31.3%) had a primary school education. A total of 1576 (48.7%) participants were observed to have a monthly household income of less than 5000 Saudi Riyals (SR), while 1101 (34.0%) respondents had an income of more than 10,000 Saudi Riyals. Additionally, the majority of participants had a normal body mass index (1410, 43.5%), while (1224, 37.8%) were classified as obese at the age of 30 and above (Table 1).

**Table 1:** General characteristics of the participants. N: number.

Characteristics			N = 3238	%
Age (years)	18–30 years		2177	67.2
	31–40 years		449	13.9
	41–50 years		382	11.8
	51–60 years		230	7.1
Gender	Male		844	26.1
	Female		2394	73.9
Marital status	Married		2055	63.5
	Unmarried		1183	36.5
Nationality	Saudi		3142	97.0
	Non-Saudi		96	3.0
Region	Western region		529	16.3
	Central region		1479	45.7
	Northern region		295	9.1
	Southern region		423	13.1
	Eastern region		512	15.8
Educational level	Primary school		1015	31.3
	Middle school		53	1.6
	High school/diploma		536	16.6
	Bachelor's degree		1430	44.2
	Postgraduate		204	6.3
Occupation	Private sector employee		345	10.7
	Public sector employee		1586	49.0
	Retired		161	5.0
	Student		779	24.1
	Do not work		367	11.3
Monthly household income (SAR)		Less than 5,000 riyals	1576	48.7
		5,000-10,000 riyals	561	17.3
		More than 10,000 riyals	1101	34.0
Body mass index (BMI)		Normal (18.5 to 24.9)	1410	43.5
		Overweight (25 to 29.9)	604	18.7
		Obesity (30 and above)	1224	37.8

**Figure 1:**
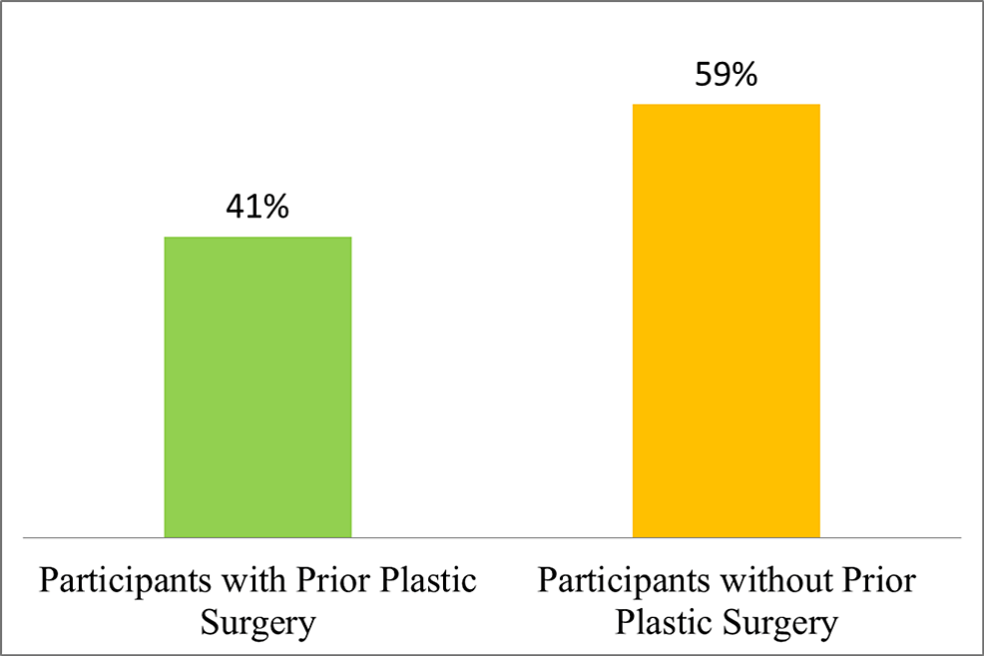
Prevalence of plastic surgery among participants in the Kingdom of Saudi Arabia.

In the Kingdom of Saudi Arabia, the prevalence of plastic surgery among participants was examined, as shown in Figure 1. Out of the total participants, a significant proportion of individuals, specifically (1328, 41%), had already undergone plastic surgery, while (1910, 59%) had not undergone any plastic surgery procedures.

Regarding the types of plastic surgery, the data reveals that the majority of participants had undergone breast augmentation surgery, specifically 1009 individuals (31.2%). Conversely, the least common type of surgery reported among the participants was dermal injections, such as collagen and fat, with only a very small percentage, 0.1%, having undergone this procedure (Table 2).

**Table 2:** Trends of plastic surgery utilization among individuals in the Kingdom of Saudi Arabia. PS: plastic surgery; N: number.

Types of plastic surgery (PS)	N = 1517	%
Facelift	24	0.7
Rhinoplasty (nose reshaping)	43	1.3
Otoplasty (ear reshaping)	8	0.2
Lip augmentation	43	1.3
Cleft lip and palate repair	14	0.4
Oral and dental procedures	61	1.9
Breast augmentation	1009	31.2
Breast reconstruction	8	0.2
Gynecomastia surgery (male breast reduction)	7	0.2
Breast lift	8	0.2
Tummy tuck (abdominoplasty)	26	0.8
Liposuction of the abdomen	18	0.6
Hand or upper limb procedures	8	0.2
Dermabrasion	5	0.2
Dermal injections (collagen/fat)	3	0.1
Dermal injections (botox/fillers such as Restylane and Radiesse)	47	1.5
Chemical peel (glycolic peel/laser peel)	8	0.2
Scar or tattoo removal from the skin	13	0.4

**Figure 2:**
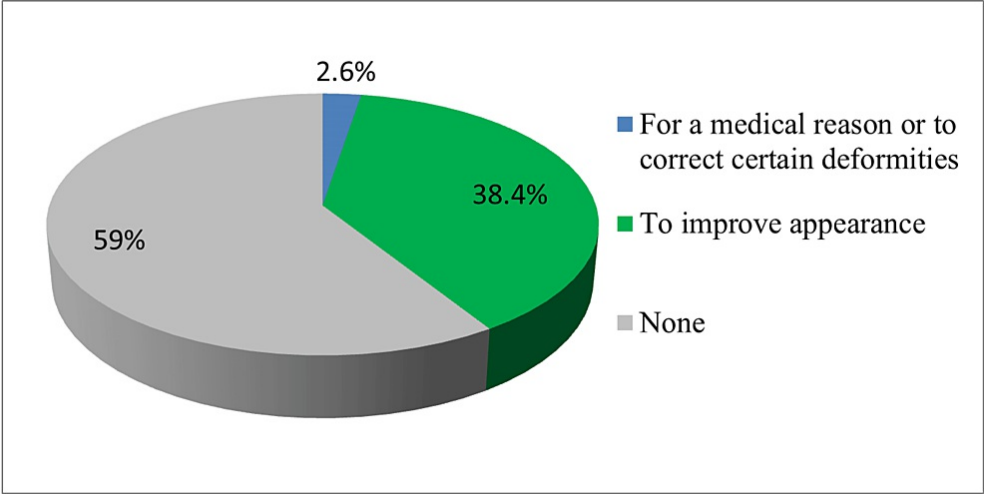
Reasons behind the trend of plastic surgery among individuals in the Kingdom of Saudi Arabia.

Out of the total sample of 3238 individuals, 1296 participants (41%) reported having undergone a cosmetic procedure. The reasons behind the prevalence of plastic surgery were explored among these participants. Among the subset of individuals who had undergone plastic surgery (1211 out of 1296), a significant majority (38.4%) stated that their primary motivation was to improve their appearance. In contrast, a smaller proportion (85 individuals, 2.6%) underwent plastic surgery for medical reasons or to address specific deformities. These findings highlight the predominance of esthetic motivations among those who had chosen to undergo plastic surgery in the study population (Figure 2).

When exploring the motivations behind participants' decisions to undergo or consider plastic surgery, the data indicates that the majority of individuals cited reasons such as enhancing their self-image (1394, 47.3%), being influenced by friends (1181, 36.5%), and being impacted by social media influencers and celebrities (1125, 34.7%). Additionally, a significant proportion (1206, 37.2%) expressed the desire to be more socially accepted, make friends, find a partner, or secure employment. Furthermore, a notable percentage (1076, 33.2%) reported seeking plastic surgery to showcase their wealth among friends, while others (1091, 33.7%) mentioned mimicking the procedures their friends had undergone. Lastly, a substantial portion of participants (1201, 37.1%) stated that they sought plastic surgery in order to conform to prevailing beauty trends (Table 3).

**Table 3:** Analyzing the trends in plastic surgery adoption or planning among individuals in the Kingdom of Saudi Arabia. N: number.

Reasons for undergoing or planning plastic surgery		N = 3238	%
Self-images	Never	1394	43.1
	No	311	9.6
	Yes	1533	47.3
Images of friends (influenced by friends)	Never	1389	42.9
	No	668	20.6
	Yes	1181	36.5
Images of celebrities (affected by social media influencers and celebrities)	Never	1406	43.4
	No	707	21.8
	Yes	1125	34.7
To be more acceptable in the community, to make friends, to get married, or to find a job	Never	1392	43.0
	No	640	19.8
	Yes	1206	37.2
To be proud between friends, indicating your wealthiest	Never	1418	43.8
	No	744	23.0
	Yes	1076	33.2
Mimic friends' work (appearance comparison)	Never	1407	43.5
	No	740	22.9
	Yes	1091	33.7
To follow the trend of beauty	Never	1402	43.3
	No	635	19.6
	Yes	1201	37.1

**Figure 3:**
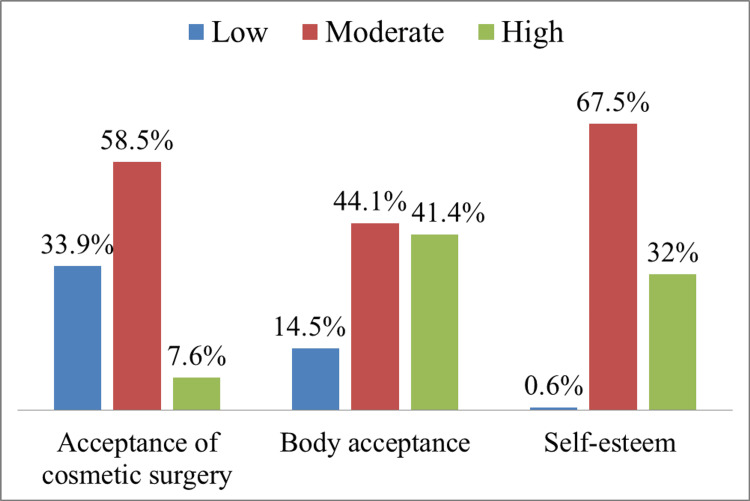
Descriptive of acceptance of cosmetic surgery, body appreciation, and self-esteem among participants in the Kingdom of Saudi Arabia.

Figure 3 provides a visual representation of the descriptive data pertaining to the acceptance of cosmetic surgery, body acceptance, and self-esteem among the participants. Specifically, the results indicate that (1341, 41.4%) of the participants displayed a high level of acceptance towards their body appearance, while (1428, 44.1%) exhibited a moderate level of acceptance. In terms of self-esteem, (1035, 32%) of the participants reported high levels, whereas the majority (2185, 67.5%) had moderate levels of self-esteem. Furthermore, a mere (245, 7.6%) of the participants demonstrated a high acceptance of cosmetic surgery, with (1895, 58.5%) displaying a moderate level of acceptance.

Table 4 presents the key characteristics of participants influencing their decision to undergo plastic surgery. Significant associations were observed between various factors and plastic surgery decisions. Age (P = 0.001) showed that 35.3% of participants aged 18-30 opted for surgery compared to 31.9% who did not. Gender (P = 0.001) revealed that 38.9% of females underwent surgery compared to 35.1% in the non-surgical group. Marital status (P = 0.001) showed that 36.5% of married participants chose surgery versus 27% who did not. Educational level (P = 0.001) indicated that 31% with primary education had surgery, while only 0.3% in the non-surgical group fell into this category. Conversely, 6.1% of those with surgery had a bachelor's degree, compared to 38% without surgery (P = 0.001). The employment sector (P = 0.001) demonstrated that 34.1% of public sector employees had surgery compared to 14.9% in other sectors. Income level and obesity status (P = 0.001) were significantly associated, with 32.6% of those with surgery reporting an income below 5000 SR, and 31.8% classified as obese. Geographically, the central region had the highest surgery rate (33.3%), followed by the western region (3.9%), with the lowest rate observed in the northern region (P = 0.001).

**Table 4:** Investigating participants' general characteristics as factors influencing the decision to undergo plastic surgery (PS). PS: plastic surgery; N: number; *: significant.

Characteristics		They underwent PS N (%)	They did not undergo PS N (%)	P
Age (years)	18–30 years	1144 (35.3)	1033 (31.9)	0.000*
	31–40 years	91 (2.8)	358 (11.1)	
	41–50 years	63 (1.9)	319 (9.9)	
	51–60 years	30 (0.9)	200 (6.2)	
Gender	Male	69 (2.1)	775 (23.9)	0.000*
	Female	1259 (38.9)	1135 (35.1)	
Marital status	Married	1182 (36.5)	873 (27.0)	0.000*
	Unmarried	146 (4.5)	1037 (32.0)	
Region	Western region	125 (3.9)	404 (12.5)	0.000*
	Central region	1077 (33.3)	402 (12.4)	
	Northern region	24 (0.7)	271 (8.4)	
	Southern region	34 (1.1)	389 (12.0)	
	Eastern region	68 (2.1)	444 (13.7)	
Educational level	Primary school	1004 (31.0)	11 (0.3)	0.000*
	Middle school	7 (0.2)	46 (1.4)	
	High school/diploma	56 (1.7)	480 (14.8)	
	Bachelor's degree	198 (6.1)	1232 (38.0)	
	Postgraduate	63 (1.9)	141 (4.4)	
Occupation	Private sector employee	86 (2.7)	259 (8.0)	0.000*
	Public sector employee	1103 (34.1)	483 (14.9)	
	Retired	27 (0.8)	134 (4.1)	
	Student	61 (1.9)	718 (22.2)	
	Do not work	51 (1.6)	316 (9.8)	
Monthly household income (SAR)	Less than 5000 riyals	1057 (32.6)	519 (16.0)	0.000*
	5000-10,000 riyals	80 (2.5)	481 (14.9)	
	More than 10,000 riyals	191 (5.9)	910 (28.1)	
Body mass index (BMI)	Normal (18.5 to 24.9)	202 (6.2)	1208 (37.3)	0.000*
	Overweight (25 to 29.9)	96 (3.0)	508 (15.7)	
	Obesity (30 and above)	1030 (31.8)	194 (6.0)	

The motivations behind the decision to undergo plastic surgery were evaluated in this study. Among the participants who underwent plastic surgery, 35.1% displayed a moderate level of acceptance towards cosmetic surgery, whereas only 23.4% of those who did not undergo the surgery exhibited a similar level of acceptance (P = 0.001). Furthermore, a mere 5.3% of the individuals who underwent plastic surgery demonstrated a high level of body appreciation, in contrast to 36.1% of those who did not undergo the surgery (P = 0.001). Additionally, only 3.8% of the participants who underwent plastic surgery reported having high self-esteem, while 28.1% of those who did not undergo the surgery exhibited high self-esteem (P = 0.001) (Table 5).

**Table 5:** Investigating potential motivators and influencing factors in the decision to undergo plastic surgery. PS: plastic surgery; N: number; *: significant.

Factors		They underwent PS	They did not undergo PS	P
Acceptance of Cosmetic Surgery Scale (ACSS)	Low acceptance	110 (3.4)	988 (30.5)	0.000*
	Moderate acceptance	1138 (35.1)	757 (23.4)	
	High acceptance	80 (2.5)	165 (5.1)	
Body Appreciation Scale (2)	Low body appreciation	95 (2.9)	374 (11.6)	0.000*
	Moderate body appreciation	1060 (32.7)	368 (11.4)	
	High body appreciation	173 (5.3)	1168 (36.1)	
Rosenberg Self-Esteem Scale	Low self-esteem	5 (0.2)	13 (0.4)	0.000*
	Moderate self-esteem	1199 (37.0)	986 (30.5)	
	High self-esteem	124 (3.8)	911 (28.1)	

## Discussion

The impact of sociocultural ideas on body image beliefs cannot be overstated. With abundant studies indicating that societal and cultural factors greatly impact body image norms, current media platforms, particularly social networking sites like Instagram and TikTok, further shape these beliefs [1-4]. When this is combined with digital appearance-altering skills, it produces potentially unrealistic beauty criteria for individuals [2,5-7].

The findings of our study, which involved a substantial sample size of 3,238 participants, provide support for these observations. Notably, a considerable proportion of participants, specifically 1353 individuals (41%), reported undergoing plastic surgery, indicating an increasing acceptance and prevalence of such procedures within our present culture. Among the various types of plastic surgery, breast augmentation emerged as the most commonly sought-after procedure, with 1009 individuals (31.2%) opting for this particular intervention. These findings align with global trends, where breast augmentation frequently ranks among the most popular cosmetic treatments [18].

Further investigation of the data reveals that 47.3% of those who underwent plastic surgery did so primarily to enhance their self-image, in contrast to only 2.6% who underwent the procedures for medically necessary or functional reasons. This finding is consistent with the aforementioned societal factors and the influence of media on individuals' perceptions of beauty. Surprisingly, a substantial proportion of participants (37.2%) sought plastic surgery to attain acceptance, often motivated by factors such as marriage and fostering new connections. This discovery sheds light on the significant societal pressures that individuals may face, emphasizing the relevance of societal norms in their decision-making processes. Concurrently, 37.1% of participants expressed their willingness to conform to prevailing beauty trends, highlighting the impact of trend-driven societal standards influenced by media portrayals and, potentially, celebrity culture. Notably, the acceptance of plastic surgery was positively associated with social media usage between 2019 and 2021, as evidenced by previous studies [8-12]. The increasing prevalence of cosmetic procedures, particularly among women, is readily apparent in Saudi Arabia, underscoring the shifting beauty paradigms within the country [19,20]. Prior research, including notable studies conducted at King Abdulaziz University Hospital, has demonstrated the beneficial effects of media exposure on attitudes toward cosmetic surgery [14].

Relationships between self-esteem, social media usage, and the acceptance of cosmetic surgery have been established, with both social media and self-esteem playing pivotal roles [8]. Furthermore, while external motivators such as the desire to avoid racial prejudice and concerns over age discrimination have been identified, internal triggers such as sadness and social anxiety have also been recognized [8]. Considering the findings of our study in conjunction with prior research, it is evident that personal and social variables significantly influence decisions regarding plastic surgery. Plastic surgery trends continue to evolve in Saudi Arabia, particularly among women.

The results of this study elucidate the significant influence of demographic factors, such as age, gender, occupation, marital status, educational level, and BMI, on individuals' decisions to undergo plastic surgery. Notably, a substantial proportion (67.3%) of participants in this study fell within the age range of 18 to 30 years. This aligns with the demographic profile of the Saudi Arabian population, where approximately 67% of individuals were under the age of 30 in 2020. This congruence with the wider population's age distribution is consistent with prior research findings. For instance, Alhusaini et al. [25], reported that the most prevalent age group among participants was under 35 years (63.1%). Similarly, Morait et al. [26], observed a similar pattern, with an average participant age of 29.37 (±9.25) years in their study.

In the present study, the age group most likely to undergo plastic surgery was found to be between 18 and 30 years old, with 52.6% of individuals in this age bracket having undergone such procedures. Conversely, the age group least likely to undergo plastic surgery was between 51 and 60 years old, with only 13% of individuals in this group having opted for plastic surgery. These findings underscore the significance of considering age as a crucial factor in analyzing the prevalence of plastic surgery within the Saudi Arabian population. Gender also emerges as a significant factor, with a higher proportion of females opting for plastic surgery compared to males. This gender disparity aligns with findings from several previous studies [25-27]. Interestingly, the current study contradicts the findings of Alhusaini et al. [25] and Morait et al. [26] regarding the relationship between marital status and plastic surgery. In this study, it was observed that married individuals were more likely to undergo plastic surgery, whereas previous studies reported a higher likelihood among single individuals. The reasons behind this discrepancy warrant further investigation.

Furthermore, while the majority of study participants hailed from various regions of Saudi Arabia, it is noteworthy that a significant majority of those who underwent plastic surgery were from the central region. While the exact reasons for this regional concentration remain unclear, it is possible that the central region boasts a higher number of plastic surgery clinics, making such procedures more accessible to residents in that area. 

Remarkably, the study findings reveal a surprising trend: individuals with lower educational levels, specifically those who completed only primary school, individuals with lower household incomes (less than 5,000 Saudi Riyals), and public sector employees, demonstrated a higher propensity for undergoing plastic surgery compared to their counterparts with higher educational levels (bachelor's degree or postgraduate) and higher household incomes (exceeding 10,000 Saudi Riyals). This observation challenges conventional assumptions and suggests that factors beyond education and income exert a substantial influence on individuals' decisions regarding plastic surgery.

Another surprising discovery is that individuals with a normal BMI exhibit a higher frequency of undergoing plastic surgery compared to those with a high BMI. This finding can be attributed to various factors that warrant consideration. Firstly, it is crucial to acknowledge that body image concerns are not limited to individuals with a high BMI. Even individuals within a healthy weight range may harbor specific body image concerns or desires for enhancement, which can serve as strong motivations for seeking plastic surgery. Moreover, it is important to recognize that plastic surgery encompasses a wide range of procedures that are not solely focused on weight-related issues. Individuals with a normal BMI may have specific interests in non-weight-related surgeries, reflecting their diverse cosmetic preferences. Their motivations for seeking plastic surgery may revolve around enhancing particular features rather than addressing weight-related concerns.

In the context of this investigation, the influence of self-esteem, body appearance, and acceptance of plastic surgery was explored, revealing that self-esteem plays a significant role in individuals' decision-making process when considering plastic surgery. The research findings indicate that a substantial proportion of participants who opted for plastic surgery exhibited moderate self-esteem (37%), whereas a minimal percentage of individuals with low self-esteem (0.2%) pursued such procedures. These results suggest that low self-esteem is not the sole driving factor behind the decision to undergo plastic surgery. In fact, previous studies consistently demonstrate a positive association between low self-esteem and the inclination to seek and accept plastic surgery [28]. Individuals with low self-esteem may perceive their physical appearance as a source of discontent or insecurity, prompting them to seek surgical interventions aimed at ameliorating perceived flaws. Furthermore, there is evidence linking the use of certain applications, such as WhatsApp and Photoshop, to lower self-esteem scores [8]. Conversely, another study uncovered a slight positive relationship between participants' self-esteem and their willingness to accept cosmetic surgery, although this correlation lacked statistical significance [13].

Nevertheless, it is noteworthy that the relationship between self-esteem and plastic surgery may not be unidirectional. Undergoing plastic surgery has the potential to positively influence self-esteem and enhance satisfaction with body image. The physical transformations achieved through surgical procedures can generate increased self-confidence and overall psychological well-being. Therefore, while low self-esteem may serve as a motivating factor for seeking plastic surgery, the procedure itself can potentially contribute to the improvement of self-esteem.

The perception of body appearance represents another significant determinant influencing individuals' decisions to undergo plastic surgery. Those who experience dissatisfaction with their body, particularly specific features or proportions, may opt for surgical interventions to address these concerns. However, our study's findings revealed that only a small subset of participants with low body esteem actually underwent plastic surgery, while the majority of individuals who pursued such procedures exhibited an average level of satisfaction with their body appearance. Consistent with previous research, there is a well-established positive correlation between body dissatisfaction and the inclination to seek plastic surgery [1]. Conversely, a separate study found no significant association between body dysmorphic disorder and the influence of facial plastic surgery [29]. The media, societal beauty standards, and social comparisons among peers often contribute to distorted perceptions of body image, compelling individuals to seek surgical alterations in order to conform to these idealized norms. The proliferation of social media platforms further amplifies these influences, as individuals are exposed to meticulously curated images showcasing seemingly flawless bodies. Consequently, the desire to attain an idealized body image can significantly impact individuals' decisions to undergo plastic surgery.

In this particular study, a notable finding was that 43% of participants expressed their reluctance to undergo plastic surgery for the purpose of gaining societal acceptance, making friends, finding a job, or getting married. The acceptance of plastic surgery within a given society or cultural context is a significant factor that influences individuals' decision-making processes. Previous research emphasizes the influence of cultural factors, social norms, and general perceptions surrounding plastic surgery on its acceptance. It has been observed that increased engagement with social media platforms correlates with a higher consideration of cosmetic surgery [8]. Cultures that prioritize physical appearance, youthfulness, and beauty may exhibit greater acceptance of plastic surgery. Within such societies, individuals may feel more at ease openly discussing and pursuing plastic surgery in order to meet societal ideals. Conversely, cultures that prioritize natural beauty or hold stigmatized views toward cosmetic interventions may discourage individuals from seeking plastic surgery.

Therefore, considering the cultural context is crucial when interpreting the impact of societal acceptance on decisions related to plastic surgery. In conclusion, this research discussion highlights the influence of self-esteem, body appearance, and societal acceptance on individuals' decisions to undergo plastic surgery. These findings are consistent with previous studies and emphasize the enduring impact of these determinants.

The research on the prevalence of plastic surgery and its determinants among the population in Saudi Arabia exhibits both limitations and strengths. One notable limitation is the gender disparity in participation, with a larger number of women included in the study compared to men. This disparity can be attributed to societal and cultural factors in Saudi Arabia, where the association of plastic surgery with women is more prevalent due to its perceived connection with adornment. Men may be less inclined to participate in the study as they may perceive plastic surgery as primarily relevant to women. Despite this limitation, it is important to note that the data collection employed a snowball method, ensuring representation from all regions of the Kingdom. However, most of the participants who responded to the study were from the central region, which may impact the generalizability of the findings to other regions.

On the other hand, the research demonstrates several strengths that distinguish it from previous studies. Notably, it is the first study in the Kingdom of Saudi Arabia to explore the association of demographic factors, acceptance of surgery, self-esteem, and body appreciation with plastic surgery in 2023. This distinctive feature contributes to a more comprehensive understanding of the factors influencing plastic surgery decisions within the Saudi Arabian context. The research also stands out for its timely investigation, considering the influence of the COVID-19 pandemic, prolonged mask-wearing, and increased virtual integration through the internet and social networking sites. These factors undoubtedly shape beauty standards and may impact individuals' motivations for plastic surgery. While the study did not specifically examine the impact of social media on individuals, it delved into other variable factors, such as demographic characteristics and self-esteem, which can evolve over time and contribute to the decision-making process.

It is crucial to acknowledge the limitations mentioned, including the gender disparity in participation and the overrepresentation of participants from the central region, as these factors may influence the generalizability of the findings to the entire Saudi Arabian population. Future research could aim to address these limitations by employing more diverse sampling methods and ensuring balanced representation across regions and genders. By doing so, a more comprehensive understanding of the determinants of plastic surgery in Saudi Arabia can be achieved. Despite these limitations, the research provides valuable insights into the sociocultural dynamics and individual motivations underlying plastic surgery, contributing to the existing body of knowledge in this field.

Recommendations

Education and Awareness

Develop educational campaigns and initiatives targeting individuals of different educational levels and socioeconomic backgrounds to raise awareness about the risks, benefits, and alternatives to plastic surgery. Emphasize the importance of making informed decisions regarding cosmetic procedures.

*Financial Accessibility* 

Explore strategies to enhance the affordability and accessibility of plastic surgery for individuals with lower incomes. This could involve implementing financial assistance programs, considering insurance coverage for specific procedures, or collaborating with charitable organizations to provide discounted or subsidized services.

Psychological Evaluation and Counseling

Establish comprehensive protocols for the psychological evaluation of individuals contemplating plastic surgery. Conduct assessments to understand their motivations, body image concerns, and mental well-being, ensuring that they have realistic expectations and are mentally prepared for the procedure. Offer counseling services before and after surgery to support individuals in dealing with the emotional and psychological aspects of the process.

*Research and Data Collection* 

Encourage further research to gain a deeper understanding of the societal and cultural factors influencing the demand for plastic surgery in Saudi Arabia. Collect comprehensive data on patient demographics, motivations, and outcomes to inform evidence-based policies and guidelines.

*Conducting Longitudinal Research* 

Conduct longitudinal studies to assess the long-term effects and satisfaction levels of individuals who have undergone plastic surgery. Such research can provide valuable insights into the psychological, social, and physical outcomes of these procedures, as well as the sustainability of their impact over time. Additionally, longitudinal studies can explore potential disparities in access to and outcomes of plastic surgery based on socioeconomic factors, education levels, and geographic regions.

## Conclusions

In summary, this study highlights the widespread prevalence of plastic surgery in Saudi Arabia and sheds light on the factors that drive individuals to choose such procedures. The primary motivation for undergoing plastic surgery is to improve one's appearance rather than a medical necessity. It is noteworthy that individuals with varying educational and income levels have pursued plastic surgery, with a significant number having completed only primary education and belonging to low-income groups. This suggests the influence of personal motivations and financial circumstances on the decision to undergo plastic surgery, as well as the role of scientific knowledge in shaping these choices.
